# Tailored Risk Stratification in Severe Mitral Regurgitation and Heart Failure Using Supervised Learning Techniques

**DOI:** 10.1016/j.jacadv.2022.100063

**Published:** 2022-08-26

**Authors:** Gregor Heitzinger, Georg Spinka, Suriya Prausmüller, Noemi Pavo, Varius Dannenberg, Carolina Donà, Matthias Koschutnik, Andreas Kammerlander, Christian Nitsche, Henrike Arfsten, Stefan Kastl, Guido Strunk, Martin Hülsmann, Raphael Rosenhek, Christian Hengstenberg, Philipp E. Bartko, Georg Goliasch

**Affiliations:** a Department of Internal Medicine II, Medical University of Vienna, Vienna, Austria; b Complexity Research, Vienna, Austria; c Herzzentrum Währing, Vienna, Austria

**Keywords:** heart failure, HFrEF, HFmrEF, HFpEF, machine learning, secondary mitral regurgitation, supervised learning

## Abstract

**Background:**

Secondary mitral regurgitation (sMR) in the setting of heart failure (HF) has considerable impact on quality of life, HF rehospitalizations, and mortality. Identification of high-risk cohorts is essential to understand disease trajectories and for risk stratification.

**Objectives:**

This study aimed to provide a structured decision tree–like approach to risk stratification in patients with severe sMR and HF.

**Methods:**

This observational study included 1,317 patients with severe sMR from the entire HF spectrum. Clinical, echocardiographic, and laboratory data were extracted for all patients. The primary end point was all-cause mortality. Survival tree analysis, a supervised learning technique, was applied to identify patient subgroups at risk of mortality and further stratified by HF subtype (preserved, mildly reduced, and reduced ejection fraction).

**Results:**

Using supervised learning (survival tree method), 8 distinct subgroups were identified that differed significantly in long-term survival. Subgroup 7, characterized by younger age (≤66 years), higher hemoglobin (>12.7 g/dL), and higher albumin levels (>40.6 g/L) had the best survival. In contrast, subgroup 5 displayed a 20-fold risk of mortality (hazard ratio: 20.38 [95% CI: 10.78-38.52]); *P* < 0.001 and had older age (>68 years), low serum albumin (≤40.6 g/L), and higher NT-proBNP levels (≥9,750 pg/mL). Unique subgroups were further identified for each type of HF subtypes.

**Conclusions:**

Supervised machine learning reveals heterogeneity in the sMR risk spectrum, highlighting the clinical variability in the population. A decision tree–like model can help identify differences in outcomes among subgroups and can help provide tailored risk stratification.

Secondary mitral regurgitation (sMR) in the setting of heart failure (HF) is frequent and is a central cause of morbidity and mortality.^1^, ^2^, ^3^ Severe sMR is specifically relevant because therapeutic strategies can improve prognosis.^4^^,^^5^ Adequate treatment relies on patient selection and risk stratification. Recently, 2 randomized trials have shown the importance of patient selection specifically for patients with severe sMR and reduced ejection fraction.^4^^,^^6^^,^^7^ Previous investigations from our group demonstrated a consistent adverse effect of severe sMR on survival in all HF subtypes (heart failure with preserved ejection fraction [HFpEF], heart failure with mildly reduced ejection fraction [HFmrEF], and heart failure with reduced ejection fraction [HFrEF]) in comparison to patients with HF and no/mild sMR. Although the effect on outcome was most pronounced in patients with HFmrEF, excess mortality was present in all HF subtypes in short- and long-term follow-up, thereby implying that other features than the HF subtype might be of central importance in the disease process.^3^

A high burden of comorbidities and systemic involvement suggest severe sMR and HF are a complex clinical entity.^3^ The results of the COAPT and MITRA-FR studies have mainly been interpreted in terms of left ventricular (LV) size and function.^4^^,^^6^ These anatomical and functional substrates of sMR are important but only partially explain the prognosis in sMR.^8^ The impact of individual risks, systemic factors, and comorbidities are less well studied but are essential for understanding prognosis, improving personalized risk stratification, and may be helpful when identifying therapeutic targets.

We therefore aimed to investigate the association between a comprehensive and readily available set of clinical, echocardiographic, and laboratory variables and outcome in HF patients with severe sMR. Furthermore, we aimed to provide a comprehensive and structured decision tree–like guide to risk stratification using supervised machine learning methods. To further improve risk stratification, we also applied this structured approach to each HF subtypes (HFpEF, HFmrEF, and HFrEF).

## Methods

### Study population

This is an observational cohort study of patients with sMR and HF identified from the Medical University of Vienna’s longitudinal health records and the institutional echocardiography database seen between 2010 and 2020. HF was diagnosed according to guidelines criteria^9^ and subclassified into HF with preserved (LV ejection fraction [LVEF] ≥50%; HFpEF), mildly reduced (LVEF 40%-49%; HFmrEF), and reduced ejection fraction (LVEF <40%; HFrEF). The diagnostic algorithm also included signs and symptoms of HF, natriuretic peptides, and echocardiographic features, such as LV hypertrophy, left atrial enlargement, or diastolic dysfunction. The diagnosis of severe sMR was based on echocardiographic assessment. Exclusion criteria included any evidence of significant primary regurgitant or stenotic mitral valve disease. Primary mitral valve disease included mitral valve prolapse, flail leaflet, rheumatic valve disease or more than moderate mitral annular calcification. In addition, patients with significant aortic or pulmonary stenosis or regurgitation and primary tricuspid valve disease were not included. From 52,995 initial echocardiographic records and 13,223 HF patients, 1,317 patients with severe sMR fulfilled our inclusion criteria.

### Echocardiographic assessment

Every patient had a comprehensive transthoracic echocardiographic examination, and those who had focused or incomplete examinations were excluded. Commercially available equipment was used, and board-certified physicians interpreted the images. Examinations and interpretation were performed as recommended by current guidelines.^10^^,^^11^ Cardiac morphology, including left and right atrial and ventricular dimensions, was assessed in standard 4- and 2-chamber views. LV function was calculated manually by biplane Simpson method, and right heart function was assessed semiquantitatively. sMR was identified by either atrial or mitral annular dilation and/or leaflet restriction due to LV remodeling and graded according to guidelines.^2^ Tricuspid regurgitation (TR) was assessed qualitatively, and systolic pulmonary artery pressure was calculated by adding the peak TR systolic gradient to the estimated central venous pressure. All echocardiographic examinations were recorded according to a structured standard protocol and read by echocardiogram board–certified physicians. In addition, echocardiographic data reliability was confirmed by resampling of 196 (15%) patients and calculation of interobserver and intraobserver consistency for etiology, acuity, and severity of sMR. The interobserver and intraobserver sample was picked randomly (R implementation) (Supplemental Table 1) but proportional to the HF subgroups size.

### Clinical and laboratory variables

Clinical and laboratory data were recorded at the time of the first echocardiographic examination. Clinical parameters of interest were age, sex, body mass index, and common clinical comorbidities, such as coronary artery disease or hypertension (full list presented in Table 1). Routine laboratory parameters were analyzed from venous blood samples according to the local laboratory’s standard protocol. Relevant variables included complete blood counts, markers of renal function, liver enzymes, total cholesterol, and brain natriuretic peptide. Both comorbidities and laboratory values were extracted from the Medical University of Vienna’s patient database.

**Table 1:** Baseline Clinical, Echocardiographic, and Laboratory Parameters of Patients With Severe Secondary Mitral Regurgitation Across the Heart Failure Spectrum

	All Patients (N = 1,317)	Heart Failure With Preserved Ejection Fraction (n = 331)	Heart Failure With Mildly Reduced Ejection Fraction (n = 330)	Heart Failure With Reduced Ejection Fraction (n = 656)	*P* Value
Clinical characteristics					
Age, y	71 (61-78)	74 (64-79)	74 (65-80)	68 (58-77)	**<0.001**
Male	792 (60%)	132 (40%)	201 (61%)	459 (70%)	**<0.001**
Body mass index, kg/m^2^	26 (24-29)	26 (23-30)	27 (24-29)	26 (23-29)	**0.032**
Comorbidities					
Hypertension	677 (51%)	154 (47%)	196 (59%)	327 (50%)	**0.002**
Hyperlipidemia	366 (28%)	74 (22%)	111 (34%)	181 (27%)	**0.005**
Diabetes	276 (21%)	56 (17%)	77 (23%)	143 (22%)	0.097
Coronary artery disease	641 (49%)	105 (32%)	175 (53%)	361 (55%)	**<0.001**
Atrial fibrillation	492 (37%)	138 (42%)	139 (42%)	215 (33%)	**0.003**
COPD	175 (13%)	37 (11%)	41 (12%)	97 (15%)	0.250
Cerebral vascular disease	223 (19%)	50 (26%)	64 (19%)	109 (17%)	**0.011**
Peripheral vascular disease	300 (23%)	72 (22%)	94 (29%)	134 (20%)	**0.015**
Echocardiographic characteristics					
Left ventricular end-diastolic diameter, mm	53 (47-60)	46 (42-50)	51 (47-56)	58 (52-64)	**<0.001**
Left atrial diameter, mm	65 (60-71)	64 (59-70)	64 (59-71)	65 (60-71)	0.088
Right ventricular end-diastolic diameter, mm	36 (32-40)	34 (31-38)	35 (32-39)	37 (33-42)	**<0.001**
Right atrial diameter, mm	61 (56-68)	60.0 (55-68)	61 (56-68)	62 (56-68)	0.322
Left ventricular dysfunction					**<0.001**
Absent	287 (21.8%)	287 (86.7%)	0 (0%)	0 (0%)	
Mild	157 (11.9%)	44 (13.3%)	113 (34.2%)	0 (0%)	
Moderate	217 (16.5%)	0 (0%)	217 (65.8%)	0 (0%)	
Severe	656 (49.8%)	0 (0%)	0 (0%)	656 (100%)	
Reduced right ventricular function (>moderate)	568 (44%)	52 (16%)	102 (31%)	414 (64%)	**<0.001**
Tricuspid regurgitation					0.194
Mild	331 (25.3%)	83 (25.2%)	92 (28.0%)	156 (23.9%)	
Moderate	488 (37.3%)	118 (35.9%)	32 (40.1%)	238 (36.5%)	
Severe	491 (37.5%)	128 (38.9%)	105 (31.9%)	258 (39.6)	
Pulmonary artery pressure (mm Hg)	53.6 (46-64.8)	53.6 (43.6-64.8)	53.6 (46-64.8)	53.6 (46-64.0)	0.995
Laboratory measurements					
Hemoglobin, g/dL	12.3 (11-14)	11.7 (10-13)	12 (10-14)	13 (11-14)	**<0.001**
Platelets	216 (174-273)	215 (174-264)	226 (179-287)	214 (173-270)	0.175
White blood cell count, g/L	7.7 (6.2-9.4)	7.3 (5.8-9.2)	7.7 (6.2-9.8)	7.8 (6.4-9.4)	**0.028**
Creatinine, mg/dL	1.1 (0.9-1.5)	1.0 (0.9-1.4)	1.1 (0.9-1.4)	1.2 (0.9-1.5)	**<0.001**
Blood urea nitrogen, mg/dL	21.3 (15.5-31.3)	19.7 (13.8-28.5)	20.9 (16.0-29.7)	22.6 (16.6-33.7)	**<0.001**
Albumin, g/L	37.9 (33.5-41.6)	38.4 (33.3-42.3)	38.2 (33.8-40.9)	37.6 (33.5-41.3)	0.410
Bilirubin, mg/dL	0.7 (0.5-1.1)	0.6 (0.4-0.8)	0.7 (0.5-0.9)	0.8 (0.6-1.3)	**<0.001**
Aspartate transaminase, U/L	27.0 (21.0-38.0)	26.0 (20.0-36.0)	27.5 (21.0-37.8)	28.0 (22.0-40.2)	**0.033**
Alanine transaminase, U/L	24.0 (17.0-41.0)	22.0 (15.0-33.0)	24.0 (17.0-37.8)	27.0 (18.0-46.0)	**<0.001**
γ-Glutamyl transferase, U/L	58.0 (33.0-115.0)	48.5 (27.0-91.2)	52.5 (30.0-88.5)	75.0 (38.0-132.0)	**<0.001**
Total cholesterol, mg/dL	151.0 (120.0-183.0)	158.0 (129.0-196.0)	154.0 (122.2-192.0)	145.0 (115.0-173.0)	**<0.001**
NT-proBNP, pg/mL	3699.5 (1703.2-8223.5)	1996.0 (947.1-4089.5)	3627.0 (1753.0-6895.0)	5234.5 (2522.8-11764.2)	**<0.001**
MV intervention within observation period					
MV repair	91 (6.9%)	39 (11.8%)	30 (9%)	22 (3.4%)	**<0.001**
MV replacement	62 (4.7%)	42 (12.7%)	15 (4.5%)	5 (0.8%)	**<0.001**
Transcatheter MV repair	47 (3.6%)	15 (4.5%)	7 (2%)	25 (3.8%)	**0.222**
Device therapy and transplant					
Cardiac resynchronization therapy	10 (0.8%)	0 (0%)	1 (0.3%)	9 (1.4%)	**0.028**
Left ventricular assist device	1 (0.07%)	1 (0.3%)	0 (0%)	0 (0%)	
Heart transplant	29 (2.2%)	0 (0.0%)	2 (0.6%)	27 (4.1%)	**<0.001**

Values are median (interquartile) or n (%). **Bold** values indicate statistical significance.

COPD = chronic obstructive pulmonary disease; HFmrEF = heart failure with mildly reduced ejection fraction (40%-50%); HFpEF = heart failure with preserved ejection fraction (>50%); HFrEF = heart failure with reduced ejection fraction (<40%); NT-proBNP = N-terminal pro brain-type natriuretic peptide.

### Outcomes

The primary end point was all-cause mortality and obtained by retrieval query from the Austrian national death registry. This study was approved by the Medical University of Vienna’s ethics committee.

### Internal and temporal validation

All patients in the study cohort were allocated to a derivation or validation cohort. Patients were split using random sampling with 2 conditions (see Supplemental Table 1 for R package implementation). First, 70% of the patients were assigned to the derivation cohort, whereas the remaining 30% served as the validation cohort, and second, the splitting procedure was stratified by HF subtype to ensure equal representation of each HF subtype. All main analyses (ie, univariate analysis, bootstrap selection, and survival tree analysis) were performed on the derivation data set, and results were then internally validated on the validation cohort. In addition, we implemented temporal validation to ensure consistency of sMR diagnosis and applicability of identified subgroups over a long period. Patients were therefore pooled into 2 cohorts stratified by inclusion year in an alternating pattern. We also applied our below outlined methodology on each HF subtype separately with the learning sample derived from the whole study cohort. Because of sample size limitations, we choose to abstain from internal validation in the subgroup analysis (HFpEF, HFmrEF, and HFrEF).

### Statistical analysis

Continuous data are presented as median and interquartile range, and categorical data are presented as count and percent. Data were compared with the Kruskal-Wallis test for the former and chi-square test for the latter. The results of the univariate Cox proportional hazard regression analysis were depicted in forest plots. Three models were formed, encompassing: 1) all clinical parameters; 2) all laboratory variables; and 3) all echocardiographic variables and a stepwise bootstrap resampling procedure using forward and backward selection with 500 repeats was applied to each model. This bootstrap resampling procedure^2^^,^^12^^,^^13^ was used to identify those variables, which were most frequently included in the respective final model. Variables selected in ≥85% of all repeats were kept for further analyses and used as covariates for a survival tree–based model, a form of supervised learning.^14^ Supervised learning refers to a form of machine learning, where the underlying data are labeled, and the aim is classification of observations based on the available features.^15^^,^^16^ The survival tree method applies recursive partitioning and groups patients in accordance to their survival data. The survival tree is grown by iteratively performing 2 steps at every split until a stopping criterion is met, and the tree ends in its terminal leaves (ie, the final risk groups). In the first step out of all covariates, the variable with the highest association to mortality is selected, and then as a second step, the optimal split that maximizes the survival difference for the chosen variable is assessed. These analyses are performed by means of *P* value adjusted log-rank tests and based on a permutation test framework introduced by Strasser and Weber.^17^ In our case, the stopping criterion was a minimum of 95 patients in the terminal leaves to ensure a large enough sample size for subsequent Kaplan-Meier analysis but still retain sufficient and clinically relevant subgroup differentiation. The minimum criterion for node split was defined as *P* < 0.05. The Central Illustration provides a visual reproduction of the methodology used. In a final step, we used Kaplan-Meier and univariate Cox proportional hazards regression analysis to further investigate each identified subgroup. All analyses were planned and performed in accordance with the Proposed Requirements for Cardiovascular Imaging-Related Machine Learning Evaluation (PRIME): A Checklist, recently published by Dr Sengupta et al.^18^ The checklist list is provided in Supplemental Table 2. For all analyses, a 2-sided *P* value <0.05 was considered statistically significant. SPSS 25 (IBM Corp) and the R software (R Core Team [2020], R: A language and environment for statistical computing, R Foundation for Statistical Computing) were used for all analyses. A detailed description of packages used is provided in Supplemental Table 1.

**Central Illustration:**
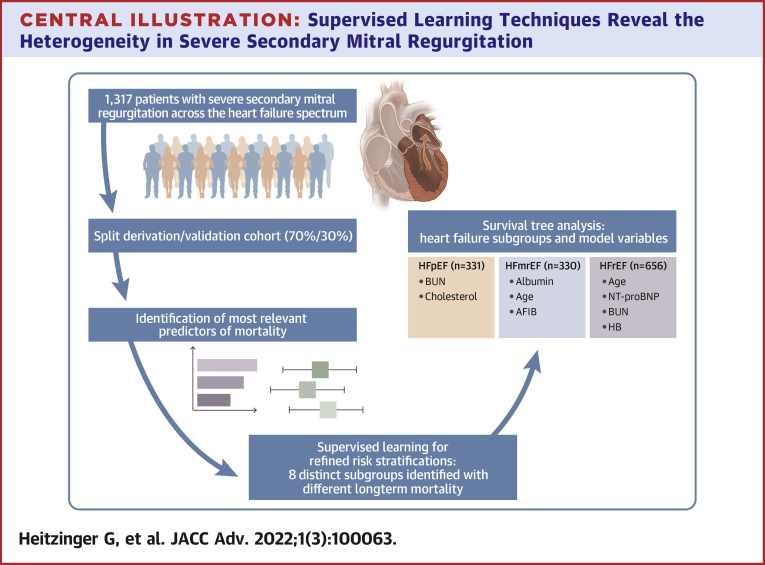
**Supervised Learning Techniques Reveal the Heterogeneity in Severe Secondary Mitral Regurgitation** The study included 1,317 patients with severe secondary mitral regurgitation and heart failure. Clinical, echocardiographic, and laboratory parameters were used to predict mortality in the derivation cohort. The most relevant predictors were investigated further using supervised learning techniques and 8 distinct subgroups could be identified, revealing a heterogenous risk spectrum in patients with severe secondary mitral regurgitation and heart failure. Additional analysis demonstrated unique risk factor profiles for each heart failure subtype. AFIB = atrial fibrillation; BUN = blood urea nitrogen; HB = hemoglobin; HFmrEF = heart failure with mildly reduced ejection fraction; HFpEF = heart failure with preserved ejection fraction; HFrEF = heart failure with reduced ejection fraction; NT-proBNP = N-terminal pro brain-type natriuretic peptide.

## Results

### Baseline characteristics of the total study population

A total of 1,317 patients (median age 71 [61-78] years) with severe sMR and HF were included in this observational study, 60% of which were male. Among those, 331 had HFpEF, 330 had HFmrEF, and 656 had HFrEF. A comprehensive overview of baseline characteristics for all HF subtypes is presented in Table 1. Overall comorbidity burden was high, as almost half of the patients had a history of CAD (n = 641, 49%), 677 (51%) had hypertension, and diabetes was present in 276 (21%). LV end-diastolic diameter (*P* < 0.001) and right ventricular end-diastolic diameter (*P* < 0.001) both increased with worsening systolic function; however, atrial dimensions did not significantly differ between HF subtypes (Table 1). Right ventricular function declined with decreasing LV function (*P* < 0.001), whereas TR severity was similar (*P* = 0.194) across all HF subtypes. NT-proBNP was elevated with a median of 3,699.5 (IQR: 1,703.2-8,223.5) pg/mL in the total study cohort and differed within the HF spectrum (*P* < 0.001). Within the observation period, 91 (6.9%) patients underwent surgical mitral valve (MV) repair, 62 (4.7%) underwent MV replacement, and 47 (3.6%) underwent transcatheter MV repair.

### Echocardiographic data reliability and intraobserver/interobserver variability

Echocardiographic data resampling confirmed consistent etiology, acuity, and severity of sMR. These data are presented in Supplemental Table 3. Intraclass correlation coefficient and Kappa statistics showed excellent correlations in accordance with recommendations from the American Society of Echocardiography,^19^ and in addition, Bland-Altman plots (Supplemental Figure 1) show good agreement of vena contracta width measurements and indicate no systemic bias. Moreover, the raw data is presented in Supplemental Table 5.

### Derivation and validation cohort characteristics

Conditional random sampling of individual patient data allocated 923 patients to the derivation cohort and 394 to the validation data set. Accordingly, strata were equally weighted in the initial and the split data sets (25% HFpEF, 25% HFmrEF, and 50% HFrEF). Baseline characteristics according to the respective cohort are presented in Supplemental Table 4 and showed equal data distribution without evidence of systematic bias.

### Risk phenotyping of patients with severe sMR and HF in the derivation cohort

The median survival time of patients in the derivation cohort was 5.4 years, in which 371 patients died. In the first step, the association between all recorded parameters and outcome by univariate Cox regression analysis (Supplemental Figure 2) was investigated. We then used a bootstrap resampling procedure to identify the most comprehensive set of variables associated with adverse outcome among: 1) all clinical parameters; 2) all echocardiographic variables; and 3) all laboratory variables (Supplemental Figure 3). Within the clinical parameters, age and peripheral artery disease (PAD) were selected most frequently. In the echocardiographic model, LV function and LV end-diastolic diameter were the most significant predictors, and among laboratory variables, blood urea nitrogen (BUN), creatinine, and albumin were found to be the most frequent predictors.

### Survival tree–based model for patients with sMR and HF in the derivation cohort

**Figure 1:**
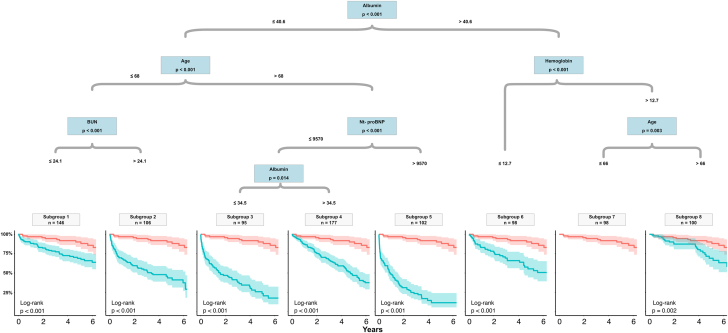
**Survival Tree Analysis for Patients With Severe Secondary Mitral Regurgitation and Heart Failure (Derivation Cohort)** Subgroups of patient with severe sMR and heart failure identified by Survival tree analysis. Predictors, cutoffs, and the according Kaplan-Meier curve are depicted in tree nodes and final leaves. Subgroup 7 had the most favorable survival and served as the reference group **(red color)**. BUN = blood urea nitrogen; NT-proBNP = N-terminal pro brain-type natriuretic peptide.

**Figure 2:**
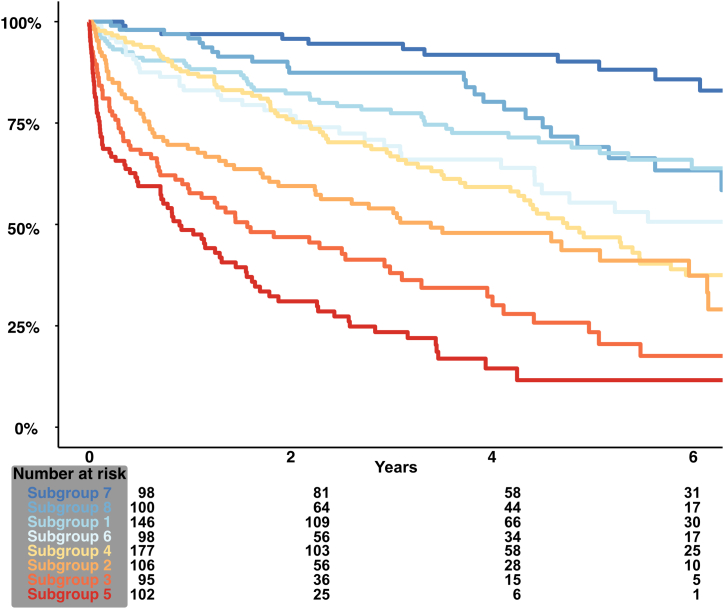
**Kaplan-Meier Survival Curves Stratified According to Supervised Learning Derived Subgroups (Derivation Cohort)** Kaplan-Meier curves for each subgroup and according numbers of patients at risk. Subgroups are color coded. Subgroup 7 **(dark blue)** displaying the lowest risk of mortality and subgroup 5 **(dark red)** depicting excessive risk of death.

All prior selected variables were further analyzed by recursive partitioning into 8 distinct risk groups that significantly differ in their long-term survival. Predictors, cutoffs, and the according Kaplan-Meier curve for each subgroup are depicted in Figure 1. Patients with the most favorable survival were allocated to subgroup 7, characterized by several splits. These include a younger age ≤66 years, hemoglobin >12.7 g/dL, and albumin >40.6 g/L and resulted in an estimated survival of 97% after 1 year and 85% after 6 years. In comparison, the patient subset with the highest mortality was subgroup 5, with an estimated survival of 48% after 1 year and 11% after 6 years consisting of older patients (age >68 years) with hypoalbuminemia (≤40.6 g/L) and highly elevated NT-proBNP (9,570 pg/mL). The remaining subgroups and their respective cutoffs are visualized using a survival tree in Figure 1. Survival analysis by Kaplan-Meier estimates is presented in Figure 2 and color coded according to survival. With subgroup 7 as reference, the according hazard ratios (HRs) for every subgroup were calculated in Table 2. Subgroup 8 with the second-best survival rate already displayed a nearly 3-fold risk increase (HR: 2.72 [95% CI: 1.33-5.55], *P* = 0.006). Patients in subgroup 5 had the highest risk of mortality, displaying a near 20-fold increase in the risk of mortality (HR: 20.38 [95% CI: 10.78-38.52]; *P* < 0.001).

**Table 2:** Subgroups Identified by Survival Tree Analysis in the Derivation and Validation Cohorts

Subgroup	Subgroup Criteria	Derivation Cohort (n = 923)	*P* Value	Validation Cohort (n = 394)	*P* Value
Subgroup 7	Albumin >40.6/HB >12.7/age ≤66	Reference		Reference	
Subgroup 8	Albumin >40.6/HB >12.7/age >66	2.72 (1.33-5.55)	0.006	12.12 (1.58-93.25)	0.017
Subgroup 1	Albumin ≤40.6/age ≤68/BUN ≤24.1	3.14 (1.62-6.09)	0.001	10.74 (1.43-80.70)	0.021
Subgroup 6	Albumin >40.6/HB ≤12.7	4.68 (2.38-9.20)	<0.001	16.13 (2.15-121.24)	0.007
Subgroup 4	Albumin ≤40.6/age >68/NT-proBNP ≤9,570/albumin >34.5	5.74 (3.05-10.80)	<0.001	15.87 (2.16-116.84)	0.007
Subgroup 2	Albumin ≤40.6/age ≤68/BUN >24.1	8.27 (4.34-15.76)	<0.001	20.3 (2.71-151.76)	0.003
Subgroup 3	Albumin ≤40.6/age >68/NT-proBNP ≤9,570/albumin ≤34.5	13.12 (6.9-24.94)	<0.001	27.38 (3.63-206.61)	0.001
Subgroup 5	Albumin ≤40.6/age >68/NT-proBNP >9,570	20.38 (10.78-38.52)	<0.001	47.23 (6.42-347.52)	<0.001

Values are HR (95% CI) unless otherwise indicated.

BUN = blood urea nitrogen (mg/dL); HB = hemoglobin (g/dL); NT-proBNP = N-terminal pro brain-type natriuretic peptide (pg/mL).

### Internal and temporal validation of survival tree model in the validation cohort

Subgroups with respective cutoffs identified by survival tree analysis based on the derivation cohort were validated on the remaining 394 patients. Kaplan-Meier analysis (Supplemental Figure 4) yielded consistent results and showed similar differentiation of subgroups. Cox proportional hazard analysis, depicted in Table 2, further supported the robustness of these results. To verify the applicability of our 8 risk groups on long-term data, temporal validation was performed. These results are depicted in Supplemental Figure 5 and show overall good consistency of subgroups.

### Predictors of mortality and survival among HF subtypes in the total study cohort

Univariate Cox regression analysis for each clinical, echocardiographic, and laboratory predictor according to HF subtype is depicted in Supplemental Figure 6. Supplemental Figure 7 shows the results of stepwise bootstrap selection.

**Figure 3:**
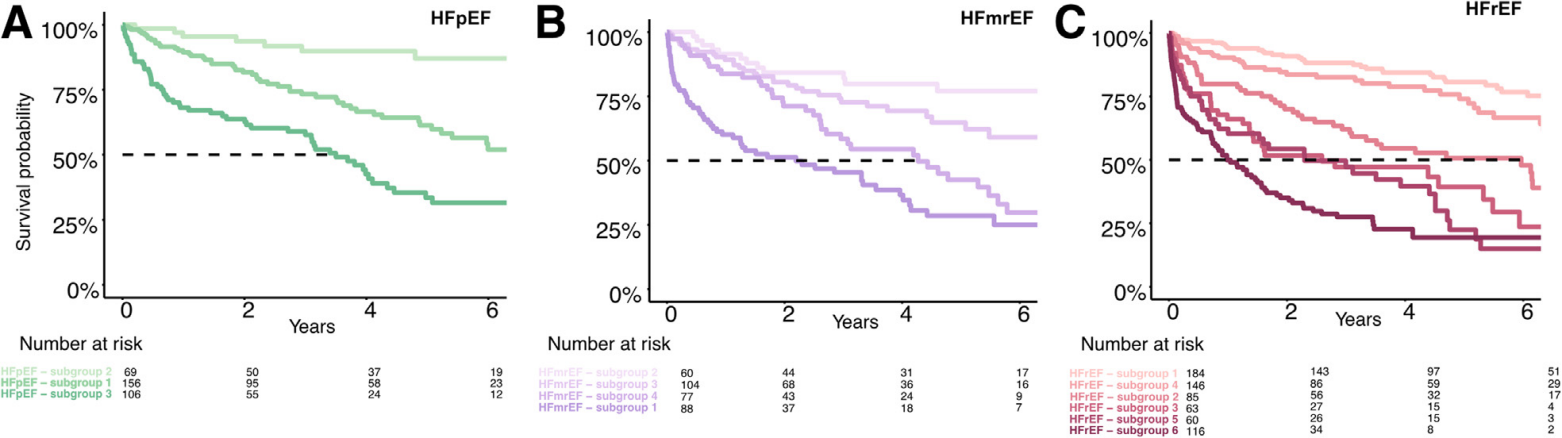
**Kaplan-Meier Survival Curves Stratified According to Heart Failure Subtypes (Subgroup Analysis)** Kaplan-Meier curves for each heart failure subtype depicting the survival of every subgroup identified by survival tree analysis. Subgroups with excessive risk of mortality are color coded in darker shades. **A** visualizes HFpEF patients **(green)**, **B** depicts HFmrEF patients **(purple)**, and **C** consists of HFrEF population **(red)**. HFmrEF = heart failure with mildly reduced ejection fraction; HFpEF = heart failure with preserved ejection fraction; HFrEF = heart failure with reduced ejection fraction.

In HFpEF, patients’ age and female sex were selected most frequently in the clinical model, and bilirubin, BUN, cholesterol, and albumin were selected most frequently in the laboratory model (Supplemental Figure 7). The survival tree analysis (Supplemental Figure 8) identified 3 separate subgroups. HFpEF patients with BUN >24.7 mg/dL had the lowest survival of 31% at 6 years. A further split was selected with cholesterol, where subgroup 2 with higher total cholesterol and better kidney function had the lowest risk of mortality. The details are shown on the Kaplan-Meier curves and corresponding HRs in Figure 3A and Table 3. Impaired kidney function in HFpEF subgroup 3 was associated with a nearly 8-fold risk hazard increase in comparison to subgroup 2 (HR: 8.03 [95% CI: 3.83-16.85]; *P* < 0.001).

**Table 3:** Subgroups Identified by Survival Tree Analysis According to Heart Failure Subtypes

Heart Failure Subtype	Model Variables	HR (95% CI)	*P* Value
HFpEF (n = 331)			
Subgroup 2	BUN ≤24.7/cholesterol >186	Reference	
Subgroup 1	BUN ≤24.7/cholesterol ≤186	3.55 (1.68-7.50)	**<0.001**
Subgroup 3	BUN >24.7	8.03 (3.83-16.85)	**<0.001**
HFmrEF (n = 330)			
Subgroup 2	Albumin >35.2/age ≤65	Reference	
Subgroup 3	Albumin >35.2/age >65/atrial fibrillation absent	1.88 (0.97-3.66)	0.062
Subgroup 4	Albumin >35.2/age >65/atrial fibrillation present	3.50 (1.83-6.69)	**<0.001**
Subgroup 1	Albumin ≤35.2	5.70 (3.05-10.66)	**<0.001**
HFrEF (n = 656)			
Subgroup 1	Age ≤65/NT-proBNP ≤5,938	Reference	
Subgroup 4	Age >65/NT-proBNP ≤10,100/BUN ≤28.9/HB >12.3	1.65 (1.03-2.62)	**0.036**
Subgroup 2	Age ≤65/NT-proBNP >5,938	3.31 (2.10-5.20)	**<0.001**
Subgroup 3	Age >65/NT-proBNP ≤10,100/BUN ≤28.9/HB ≤12.3	5.30 (3.30-8.49)	**<0.001**
Subgroup 5	Age >65/NT-proBNP ≤10,100/BUN >28.9	6.43 (4.06-10.20)	**<0.001**
Subgroup 6	Age >65/NT-proBNP >10,100	9.34 (6.21-14.06)	**<0.001**

**Bold** values indicate statistical significance.

BUN = blood urea nitrogen (mg/dL); HB = hemoglobin (g/dL); HFmrEF = heart failure with mildly reduced ejection fraction (40%-50%); HFpEF = heart failure with preserved ejection fraction (>50%); HFrEF = heart failure with reduced ejection fraction (<40%); NT-proBNP = N-terminal pro brain-type natriuretic peptide (pg/mL); PAD = peripheral artery disease.

In HFmrEF, the most frequently selected variables were atrial fibrillation, body mass index, LV end-diastolic diameter, albumin, and aspartate transaminase (Supplemental Figure 7). In survival tree analysis, HFmrEF subgroup 1, defined by hypoalbuminemia (≤35.2 g/L), had the worst survival at 25% after 6 years. The remaining subgroups were stratified according to age and presence of atrial fibrillation. Subgroup 2 with younger patients and no hypoalbuminemia had the best survival. The survival tree is visualized in Supplemental Figure 9, and the risk of mortality for each subgroup is presented in Table 3. In comparison to subgroup 2, patients with hypoalbuminemia in subgroup 1 had a significantly higher mortality (HR: 5.7 [95% CI: 3.05-10.66]; *P* < 0.001).

In HFrEF, age and PAD were the most frequently selected clinical variables, and LV end-diastolic diameter was the most frequently selected echocardiographic variable. NT-proBNP was the most selected laboratory variable, followed by aspartate transaminase, hemoglobin, and BUN. The survival tree in HFrEF patients was mainly shaped by NT-proBNP. Subgroup 1, consisting of younger patients with lower NT-proBNP levels, had the most favorable survival prognosis. Mortality was highest in older patients with excessive NT-proBNP levels in subgroup 6. The partitioning algorithm also determined additional subgroups, which were defined by renal function and hemoglobin levels. Apart from subgroup 1, survival was severely impaired in all patients (Supplemental Figure 10). In reference to subgroup 1, the risk of mortality saw a near 9-fold increase in subgroup 6 (HR: 9.34 [95% CI: 6.21-14.06]; *P* < 0.001). HRs for the corresponding subgroups are in Table 3.

## Discussion

In this study, we performed a unique large-scale in-depth risk analysis in patients with sMR and HF using a supervised machine learning approach. This method allowed us to examine a large relatively comprehensive set of predictive variables. The main findings included: 1) the identification of clinical, echocardiographic, and laboratory variables important for risk stratification; 2) the delineation of a heterogeneous risk spectrum in severe sMR reflecting variable systemic involvement and comorbid conditions; 3) risk stratification by survival tree analysis allows identification of patient subgroups at disproportionately high risk; and 4) internal as well as temporal validation of risk prediction.

### sMR and HF: a heterogenous spectrum

Diagnosis, management, and treatment of severe sMR are complex due to comorbidities, diverse anatomic substrates, and variable systemic involvement. Historically, HF subtypes are based on measurements of the LVEF but have subsequently been refined to also include other diagnostic criteria, such as neurohumoral activation, relevant structural heart disease, and diastolic dysfunction. We have previously shown that severe sMR is associated with excess mortality in all HF subtypes without significant differences between the subtypes,^3^ indicating that risk stratification needs to be considered in a more holistic way to differentiate subgroups with a benign prognosis and those with disproportional high risk of mortality either due to comorbid conditions or more systemic involvement. The use of survival tree analysis allowed us to delineate the heterogeneous risk spectrum (Figure 1, Central Illustration) based on commonly available clinical, echocardiographic, and laboratory parameters. We could identify high risk subgroups with a 20-fold risk of mortality compared with the group with the most favorable mortality serving as the reference. This analysis highlights that sMR is not a simple lesion but a complex syndrome with multifaceted comorbidities, diverse anatomic substrates, and variable systemic involvement. The term covers a broad patient spectrum, ranging from patients that otherwise are nearly healthy to those with disproportionately high risk of mortality (Figure 2). This heterogenous spectrum of sMR needs to be considered, and a tailored approach to risk stratification may be a valuable tool to guide therapeutic decisions.

### Risk factors for mortality in patients with severe sMR and HF

The comprehensive set of clinical, echocardiographic, and laboratory parameters in this study covers a broad range of factors and includes patients with multiorgan involvement. Among the most important predictors in our survival trees were BUN, albumin, hemoglobin, and NT-proBNP. Previous studies have shown the predictive role of these laboratory parameters in HF patients. Low serum albumin was found to be associated with adverse cardiovascular outcomes in both HFrEF and HFpEF,^19^^,^^20^ although it remains unclear whether hypoalbuminemia is the result of impaired liver function or result of hemodilution from volume overload. In severe sMR, it is probable that the latter is the case, as liver function may not yet be severely impaired. Similar to other HF cohorts,^21^ we found low hemoglobin to be associated with mortality. Anemia secondary to hemodilution is frequent in chronic HF and has a worse prognosis than true anemia.^22^^,^^23^ BUN has been repetitively shown to be a powerful predictor of mortality in HF.^24^, ^25^, ^26^ In addition, some studies even report superiority to creatinine-based markers of renal function.^25^ In the setting of severe sMR, increases in BUN may be interpreted as a marker of forward failure. Renal malperfusion due to reduced cardiac output in severe sMR stimulates neurohumoral response and vasoconstriction in the afferent arterioles, leading to increased water and urea absorption, thereby increasing BUN.^26^

In regard to echocardiographic variables, markers of LV function and LV dimension were selected by the bootstrap procedure as the most relevant predictors of mortality. These 2 parameters are important determinants of stroke volume and therefore together with the regurgitant effect of sMR, determine the forward stroke volume, and facilitate end-organ perfusion. These findings highlight the importance of both the anatomic substrates of sMR and the systemic involvement. Bootstrap selection and survival trees also included risk factors such as age, PAD, chronic obstructive pulmonary disease, or sex. The identification of these risk factors might help with clinical decision-making. For instance, in patients with very limited life expectancy and extracardiac conditions, it is recommended that a palliative approach should be considered.^27^

Interestingly, the survival tree model was characterized by laboratory and clinical parameters, despite previously established predictive usefulness of echocardiographic variables in sMR.^1^ While echocardiographic variable will continue to be important, this study highlights that, in a survival tree model, laboratory and clinical variables provide better differentiation of subgroups for all-cause mortality.

### Risk stratification in HF subtypes

Classification of HF patients into subtypes is essential for diagnosis and treatment planning. In the setting of concomitant severe sMR, however, we previously found that the HF subtype does not add prognostic information.^3^ Therefore, the current analysis helps improve our understanding of this condition by identifying other variables that impact outcome. Nevertheless, substratification into HF subtypes still holds relevance even in sMR, as survival tree analysis revealed distinct risk features of HF subtypes, allowing for a personal and individual risk stratification (Figure 3).

### Strengths and limitations

The specific strengths of this study included: 1) a comprehensive diagnosis of HF and the specific subtypes according to societal guideline criteria; 2) detailed echocardiographic diagnostic of sMR etiology and severity; 3) risk phenotyping covering all HF subtypes as well as a broad spectrum of anatomic substrates, systemic involvement, and comorbid conditions; and 4) consistent results on internal and temporal validation highlighting the reliability of the findings. Although the present data provide the most comprehensive contemporary information about risk stratification in sMR and HF, external validation might improve applicability in clinical practice. Despite clinical, echocardiographic, and laboratory information, even more data—such as detailed medical therapy, information on secondary end points, and incorporation of other imaging modalities—could further improve individual risk estimation. Our data provide results for a very specific patient population—namely, severe sMR in HF—and therefore are not generalizable to all HF patients. Also, we chose to use a single survival tree over “bagged” decision trees. Although this may affect the predictive accuracy, model interpretability is higher, and application of structured decision trees is more feasible for everyday clinical application.

## Conclusions

Machine learning (survival tree analysis) is helpful to highlight the complex and heterogenous outcomes in patients with severe sMR and HF. Using this approach, subgroups of patients with disproportionately high mortality can be identified. This phenotype-driven approach may help with personalized risk stratification and the identification of targets for optimizing patient care.

## Funding support and author disclosures

This work was supported by a grant of the 10.13039/501100002428Austrian Science Fund (FWF – identification number: KLI-818B). The authors have reported that they have no relationships relevant to the contents of this paper to disclose.

PERSPECTIVES **COMPETENCY IN PATIENT CARE AND PROCEDURAL SKILLS:** In patients with severe sMR and HF, a heterogenous risk spectrum was identified by supervised learning techniques. Patients with younger age, better renal function, and higher hemoglobin values had the most favorable survival, whereas older patients with low serum albumin and higher NT-proBNP values experience a 20-fold risk increase in mortality.

**TRANSLATIONAL OUTLOOK:** Further studies are needed to refine the therapeutic management for sMR in every HF subtype, taking into account the complex underlying heterogeneity in this population.
