# Supplementary material for: Tailored Risk Stratification in Severe Mitral Regurgitation and Heart Failure Using Supervised Learning Techniques
Source: JACC Adv. 2022 Aug 26;1(3):100063. doi: 10.1016/j.jacadv.2022.100063 (PMC11198388; doi:10.1016/j.jacadv.2022.100063)
Supplement: Supplemental Figures 1-10 and Tables 1-5 [file mmc1.docx]

**Supplemental Table 1:** R-packages and version used

| **Package** | **Version** | **Use** |
| --- | --- | --- |
| tidyverse | 1.3.1 | Data analysis, general data handling/plotting/ programming/ import/ data subsampling |
| rsample | 0.0.8 | Random sampling split with stratification/initial_split() function |
| gtsummary | 1.4.2 | Summary of baseline characteristics/ table generation |
| ggplot2 | 3.35 | Plot generation |
| tidymodels | 0.1.2 | Dataset splitting into derivation/validation cohort |
| bootStepAIC | 1.2-0 | Stepwise bootstrap resampling procedure |
| survival | 3.2-11 | Survival analysis/ Cox regression |
| partykit | 1.2-11 | Survival tree analysis/ tree plotting |
| survminer | 0.4.8 | Kaplan – Meier- analysis |
| irr | 0.84.1 | Interclass correlation coefficient; Cohen’s and Fleiss’ Kappa |

**Supplemental Table 2:** Checklist according to PRIME Guidelines

| **Section** | **Checklist item** | **Application to this paper** |
| --- | --- | --- |
| 1.1 | Describe the need for the application of machine learning to the dataset | Page 5, introduction, to provide a refined risk-stratification |
| 1.2 | Describe the objectives of the machine learning analysis | Page 5, introduction, to provide a comprehensive and structured decision-tree-like guide to risk-stratification |
| 1.3 | Define the study plan | Page 5, introduction and page 8/9, statistical analysis |
| 1.4 | Describe the summary statistics of baseline data | Table 1 |
| 1.5 | Describe the overall steps of the machine learning workflow | Graphical abstract, page 8/9 statistical analysis |
| 2.1 | Describe how the data were processed in order to make it clean, uniform, and consistent | Page 8/9 statistical analysis |
| 2.2 | Describe whether variables were normalized and if so, how this was done | No normalization was performed |
| 2.3 | Provide details on the fraction of missing values (if any) and imputation methods | Proportion of missing data was low (<1% for variables entered into the tree model) |
| 2.4 | Describe any feature selection processes applied | Feature selection after bootstrap resampling procedure, to find most important predictors. Page 8/9, statistical analysis |
| 2.5 | Identify and describe the process to handle outliers if any | NA |
| 2.6 | Describe whether class imbalance existed, and which method was applied to deal with it | Dataset was split into derivation/validation cohort, stratified by HF subgroup to ensure balanced distribution within the two cohorts |
| 3.1 | Explicitly define the goal of the analysis e.g., regression, classification, clustering | Identification of subgroups with different hazards, to refine risk-stratification, page 5 introduction, page 8/9 statistical analysis |
| 3.2 | Identify the proper learning method used (e.g., supervised, reinforcement learning etc.) to address the problem | Supervised learning was utilized, specifically survival tree analysis, page 5, introduction |
| 3.3 | Provide explicit details on the use of simpler, complex, or ensemble models | NA, no simpler tree models applicable |
| 3.4 | Provide the comparison of complex models against simpler models if possible | Limitations section, predictive accuracy of bagged tree models vs. higher interpretability of structured decision tree. |
| 3.5 | Define ensemble methods, if used | NA |
| 3.6 | Provide details on whether the model is interpretable | One of the advantages of tree structured models is high and easy interpretability |
| 4.1 | Provide a clear description of data used for training, validation, and testing | Central illustration, page 8/9 statistical analysis |
| 4.2 | Describe how the model parameters were optimized (e.g., optimization technique, number of model parameters etc.) | Trees were pruned with minimal terminal leaf size, to ensure adequate number of patients for subsequent Cox regression analysis |
| 5.1 | Provide the metric(s) used to evaluate the performance of the model | Cox regression (C-statistic) to evaluate subgroup-model performance for derivation and validation |
| 5.2 | Define the prevalence of disease and the choice of the scoring rule used | NA |
| 5.3 | Report any methods used to balance the numbers of subjects in each class | NA |
| 5.4 | Discuss the risk associated to misclassification | Strengths and limitations section, page 18/19 |
| 6.1 | Consider sharing code or scripts on a public repository with appropriate copyright protection steps for further development and non-commercial use | NA, raw data from reread is provided and a list of used packages for this analysis is presented |
| 6.2 | Release a data dictionary with appropriate explanation of the variables | Page 6/7, statistical analysis section |
| 6.3 | Document the version of all software and external libraries used | Supplementary Table 1 |
| 7.1 | Identify and report the relevant model assumptions and findings | Cox proportional hazards assumption was satisfied for all identified subgroups |
| 7.2 | If well performing models were tested on a hold-out validation dataset, detail the data of that validation set with the same rigor as that of training dataset (see section 2 above) | Cutoffs and subgroups identified by survival tree analysis were tested on validation dataset, results are displayed in Table 2 |

**Supplemental Table 3:** Intraobserver and interobserver Correlation Coefficient and agreement between categorical variables from echocardiogram review.

| **HF Subtype** | **Vena contracta width – intraobserver correlation coefficient** | **Vena contracta width – interobserver correlation coefficient** | **Cohen’s κ - Agreement original sMR severity and reader 1** | **Cohen’s κ - Agreement original sMR severity and reader 2** | **Fleiss’ κ - Agreement etiologiy of sMR between readers** |
| --- | --- | --- | --- | --- | --- |
| **HFpEF (n = 49)** | 0.85 | 0.83 | 0.91 | 0.95 | 0.92 |
| **HFmrEF (n = 49)** | 0.82 | 0.85 | 0.95 | 0.95 | 0.92 |
| **HFrEF (n = 98)** | 0.84 | 0.81 | 0.91 | 0.91 | 0.93 |

**Supplemental Table 4:** Baseline characteristics by derivation/ validation cohort stratified by heart failure subgroup

| Characteristic | Overall, N = 1,317^1^ | Derivation cohort, N = 923^1^ | Validation cohort, N = 394^1^ | p-value^2^ |
| --- | --- | --- | --- | --- |
| Heart failure subgroup |  |  |  | >0.9 |
| HFpEF | 331 (25%) | 232 (25%) | 99 (25%) |  |
| HFmrEF | 330 (25%) | 231 (25%) | 99 (25%) |  |
| HFrEF | 656 (50%) | 460 (50%) | 196 (50%) |  |
| Age, years | 71 (61 - 78) | 71 (62 - 79) | 71 (60 - 78) | 0.2 |
| Sex, male | 792 (60%) | 556 (60%) | 236 (60%) | >0.9 |
| Body mass index, kg/m2 | 26.2 (23.7 - 29.4) | 26.2 (23.5 - 29.4) | 26.3 (23.7 - 29.4) | 0.6 |
| Hypertension | 677 (51%) | 485 (53%) | 192 (49%) | 0.2 |
| Hyperlipidemia | 366 (28%) | 259 (28%) | 107 (27%) | 0.7 |
| Diabete, type II | 276 (21%) | 196 (21%) | 80 (20%) | 0.7 |
| Coronary Artery Disease | 641 (49%) | 458 (50%) | 183 (46%) | 0.3 |
| Atrial fibrillation | 492 (37%) | 348 (38%) | 144 (37%) | 0.7 |
| COPD | 175 (13%) | 123 (13%) | 52 (13%) | >0.9 |
| Cerebral vascular disease | 223 (17%) | 155 (17%) | 68 (17%) | 0.8 |
| Peripheral vascular disease | 300 (23%) | 214 (23%) | 86 (22%) | 0.6 |
| Left ventricular dysfunction |  |  |  | 0.8 |
| Absent | 287 (22%) | 206 (22%) | 81 (21%) |  |
| Mild | 157 (12%) | 106 (11%) | 51 (13%) |  |
| Moderate | 217 (16%) | 151 (16%) | 66 (17%) |  |
| Severe | 656 (50%) | 460 (50%) | 196 (50%) |  |
| Left ventricular end-diastolic diameter, mm | 53 (47 - 60) | 53 (47 - 59) | 53 (47 - 60) | 0.4 |
| Left atrial diameter, mm | 65 (60 - 71) | 65 (59 - 71) | 64 (60 - 70) | 0.8 |
| Right ventricular end-diastolic diameter, mm | 36 (32 - 40) | 36 (32 - 41) | 36 (32 - 40) | 0.9 |
| Right atrial diameter, mm | 61 (56 - 68) | 61 (56 - 68) | 61 (56 - 68) | 0.8 |
| Reduced RV function (>moderate) | 118 (9.0%) | 83 (9.0%) | 35 (9.0%) | >0.9 |
| Secondary tricuspid regurgitation |  |  |  | 0.8 |
| Mild | 331 (25%) | 227 (25%) | 104 (26%) |  |
| Moderate | 488 (37%) | 345 (38%) | 143 (36%) |  |
| Severe | 491 (37%) | 345 (38%) | 146 (37%) |  |
| Pulmonary artery pressure (mmHg) | 51 (44 - 63) | 53 (44 - 64) | 51 (43 - 62) | 0.044 |
| Hemoglobin, g/dl | 12.3 (10.6 - 13.8) | 12.3 (10.6 - 13.7) | 12.5 (10.8 - 14.0) | 0.092 |
| White blood cell count, G/l | 7.7 (6.2 - 9.4) | 7.6 (6.1 - 9.4) | 7.7 (6.2 - 9.5) | 0.5 |
| Platelets | 216.0 (174.0 - 273.0) | 217.0 (173.0 - 276.0) | 215.0 (177.0 - 267.0) | >0.9 |
| CRP | 0.9 (0.3 - 2.9) | 0.9 (0.3 - 2.9) | 0.8 (0.3 - 2.7) | 0.3 |
| Creatinine, mg/dl | 1.1 (0.9 - 1.5) | 1.1 (0.9 - 1.4) | 1.1 (0.9 - 1.5) | 0.7 |
| Blood urea nitrogen, mg/dl | 21.3 (15.5 - 31.3) | 21.2 (15.3 - 30.6) | 21.9 (15.9 - 33.0) | 0.3 |
| Albumin, g/l | 37.9 (33.5 - 41.6) | 37.8 (33.5 - 41.4) | 38.1 (33.5 - 41.6) | 0.7 |
| Bilirubin, mg/dl | 0.7 (0.5 - 1.1) | 0.7 (0.5 - 1.1) | 0.7 (0.5 - 1.1) | 0.7 |
| Aspartate transaminase, U/l | 27 (21 - 38) | 27 (21 - 37) | 28 (21 - 40) | 0.7 |
| Alanine transaminase, U/l | 24 (17 - 41) | 24 (17 - 40) | 24 (17 - 42) | 0.5 |
| Gamma-Glutamyl transferase, U/l | 58.0 (33.0 - 115.0) | 59.0 (33.0 - 115.0) | 57.0 (32.0 - 117.0) | 0.6 |
| Total cholesterol, mg/dl | 151 (120 - 183) | 151 (122 - 181) | 152 (119 - 187) | 0.8 |
| Nt-proBNP, pg/ml | 3,699.5 (1,703.2 - 8,223.5) | 3,717.5 (1,675.2 - 8,188.0) | 3,611.0 (1,773.5 - 8,417.2) | 0.8 |
| Mitral valve repair | 91 (6.9%) | 65 (7.0%) | 26 (6.6%) | 0.8 |
| Mitral valve replacement | 62 (4.7%) | 36 (3.9%) | 26 (6.6%) | 0.034 |
| Transcatheter mitral valve repair | 47 (3.6%) | 34 (3.7%) | 13 (3.3%) | 0.7 |
| Any mitral valve intervention | 193 (15%) | 130 (14%) | 63 (16%) | 0.4 |
| CRT | 10 (0.8%) | 5 (0.5%) | 5 (1.3%) | 0.2 |
| LVAD | 1 (<0.1%) | 1 (0.1%) | 0 (0%) | >0.9 |
| HTX | 29 (2.2%) | 20 (2.2%) | 9 (2.3%) | 0.9 |
| ^1^n (%); Median (IQR) | | | | |
| ^2^Pearson's Chi-squared test; Wilcoxon rank sum test; Fisher's exact test | | | | |

**Supplemental Table 5:** Raw review data file:

| **ID** | **HF - subgroup** | **Original sMR severity** | **Etiology sMR reader 1/ read 1*** | **Severity sMR reader 1/ read 1** | **Vena contracta width (mm) reader 1/ read 1** | **Jet area reader 1/ read 1** | **Etiology sMR reader 1/ read 2*** | **Severity sMR reader 1/ read 2** | **Vena contracta width (mm) reader 1/ read 2** | **Jet area reader 1/ read 2** | **Etiology sMR reader 2*** | **Severity sMR reader 2** | **Vena contracta width (mm) reader 2** | **Jet area reader 2** |
| --- | --- | --- | --- | --- | --- | --- | --- | --- | --- | --- | --- | --- | --- | --- |
| 1 | HFpEF | 3 | 0 | 3 | 7,4 | 1 | 0 | 3 | 7,2 | 0 | 0 | 3 | 7,1 | 1 |
| 2 | HFpEF | 3 | 0 | 3 | 7,3 | 1 | 0 | 3 | 7,6 | 1 | 0 | 3 | 7,7 | 1 |
| 3 | HFpEF | 3 | 1 | 3 | 7,5 | 1 | 1 | 3 | 7,6 | 1 | 1 | 3 | 7 | 1 |
| 4 | HFpEF | 3 | 0 | 3 | 7,7 | 1 | 0 | 3 | 7,4 | 1 | 0 | 3 | 7,3 | 1 |
| 5 | HFpEF | 4 | 0 | 4 | 6,8 | 1 | 2 | 4 | 6,8 | 1 | 0 | 4 | 6,5 | 1 |
| 6 | HFpEF | 3 | 0 | 3 | 7,8 | 1 | 2 | 4 | 7,9 | 1 | 0 | 3 | 8,3 | 1 |
| 7 | HFpEF | 4 | 0 | 4 | 8,1 | 1 | 2 | 4 | 8,1 | 1 | 0 | 4 | 8,3 | 0 |
| 8 | HFpEF | 4 | 2 | 4 | 8 | 1 | 1 | 4 | 7,8 | 1 | 2 | 4 | 7,8 | 1 |
| 9 | HFpEF | 3 | 0 | 3 | 6,3 | 1 | 0 | 3 | 6,7 | 1 | 0 | 3 | 6 | 1 |
| 10 | HFpEF | 3 | 2 | 3 | 7,3 | 1 | 1 | 3 | 7,4 | 1 | 2 | 3 | 7,7 | 1 |
| 11 | HFpEF | 3 | 0 | 3 | 6,5 | 0 | 0 | 3 | 6,9 | 0 | 0 | 3 | 7,1 | 0 |
| 12 | HFpEF | 3 | 0 | 3 | 7,9 | 0 | 0 | 3 | 7,6 | 0 | 0 | 3 | 7,9 | 1 |
| 13 | HFpEF | 3 | 2 | 3 | 6,7 | 1 | 2 | 3 | 7,3 | 1 | 0 | 3 | 7,3 | 1 |
| 14 | HFpEF | 3 | 2 | 3 | 7,1 | 0 | 2 | 3 | 6,7 | 1 | 2 | 3 | 7 | 0 |
| 15 | HFpEF | 4 | 0 | 4 | 8,4 | 1 | 0 | 4 | 8 | 1 | 2 | 4 | 8,5 | 1 |
| 16 | HFpEF | 4 | 2 | 4 | 8,8 | 0 | 2 | 4 | 8,1 | 1 | 2 | 4 | 9,4 | 1 |
| 17 | HFpEF | 3 | 2 | 3 | 6,8 | 0 | 2 | 3 | 6,4 | 0 | 2 | 3 | 6,7 | 9 |
| 18 | HFpEF | 4 | 2 | 4 | 7,1 | 1 | 2 | 4 | 7,4 | 0 | 2 | 4 | 7,3 | 0 |
| 19 | HFpEF | 3 | 2 | 4 | 7,9 | 0 | 0 | 3 | 8,3 | 0 | 2 | 4 | 8,5 | 0 |
| 20 | HFpEF | 3 | 0 | 3 | 7,1 | 0 | 0 | 3 | 7,1 | 0 | 0 | 3 | 6,8 | 0 |
| 21 | HFpEF | 3 | 2 | 4 | 8 | 1 | 1 | 3 | 7,8 | 1 | 2 | 3 | 8,3 | 0 |
| 22 | HFpEF | 3 | 0 | 3 | 6,7 | 0 | 0 | 3 | 6,8 | 0 | 0 | 3 | 7,1 | 0 |
| 23 | HFpEF | 3 | 2 | 3 | 7,3 | 0 | 0 | 3 | 6,6 | 0 | 2 | 3 | 7 | 0 |
| 24 | HFpEF | 4 | 0 | 4 | 7,2 | 0 | 1 | 4 | 7 | 0 | 0 | 4 | 7,3 | 0 |
| 25 | HFpEF | 3 | 0 | 3 | 7,6 | 0 | 0 | 3 | 7,4 | 0 | 0 | 3 | 7,8 | 1 |
| 26 | HFpEF | 3 | 0 | 3 | 6,7 | 0 | 0 | 3 | 6,7 | 0 | 0 | 3 | 6,3 | 0 |
| 27 | HFpEF | 3 | 0 | 3 | 7,9 | 0 | 0 | 3 | 7,5 | 0 | 0 | 3 | 7,7 | 0 |
| 28 | HFpEF | 3 | 2 | 3 | 7 | 0 | 0 | 3 | 7,4 | 0 | 2 | 3 | 7,2 | 0 |
| 29 | HFpEF | 4 | 2 | 4 | 8,3 | 1 | 0 | 4 | 8,7 | 1 | 2 | 4 | 8,4 | 1 |
| 30 | HFpEF | 3 | 0 | 3 | 7,4 | 0 | 2 | 3 | 7,3 | 0 | 0 | 3 | 6,8 | 0 |
| 31 | HFpEF | 3 | 0 | 3 | 7,2 | 1 | 0 | 3 | 6,6 | 1 | 0 | 3 | 6,8 | 1 |
| 32 | HFpEF | 4 | 0 | 4 | 8,8 | 1 | 0 | 4 | 8,8 | 1 | 0 | 4 | 8,3 | 1 |
| 33 | HFpEF | 3 | 4 | 3 | 6,8 | 0 | 0 | 3 | 7 | 0 | 4 | 3 | 7,4 | 1 |
| 34 | HFpEF | 4 | 0 | 4 | 7,4 | 1 | 0 | 4 | 7,7 | 1 | 0 | 4 | 7,8 | 1 |
| 35 | HFpEF | 4 | 4 | 4 | 7,5 | 1 | 2 | 4 | 7,5 | 1 | 4 | 4 | 7,8 | 1 |
| 36 | HFpEF | 3 | 0 | 3 | 7,1 | 0 | 0 | 3 | 7,4 | 0 | 0 | 3 | 6,8 | 0 |
| 37 | HFpEF | 3 | 0 | 3 | 7,3 | 1 | 0 | 3 | 7,4 | 1 | 0 | 3 | 7 | 0 |
| 38 | HFpEF | 4 | 0 | 4 | 9,2 | 1 | 0 | 4 | 8,8 | 1 | 0 | 4 | 9,6 | 0 |
| 39 | HFpEF | 4 | 0 | 4 | 6,4 | 0 | 0 | 4 | 6,5 | 0 | 0 | 4 | 6,6 | 0 |
| 40 | HFpEF | 3 | 0 | 3 | 7,3 | 0 | 0 | 3 | 7 | 0 | 0 | 3 | 6,8 | 0 |
| 41 | HFpEF | 3 | 1 | 3 | 7,2 | 0 | 2 | 3 | 7,5 | 0 | 1 | 3 | 7,6 | 1 |
| 42 | HFpEF | 3 | 1 | 3 | 7,1 | 0 | 2 | 3 | 7,5 | 0 | 1 | 3 | 7,4 | 1 |
| 43 | HFpEF | 4 | 2 | 4 | 7,1 | 1 | 2 | 4 | 6,7 | 1 | 2 | 4 | 7,2 | 1 |
| 44 | HFpEF | 4 | 2 | 4 | 8,7 | 1 | 0 | 4 | 9 | 1 | 2 | 4 | 8 | 1 |
| 45 | HFpEF | 4 | 2 | 4 | 6,8 | 0 | 2 | 4 | 7,2 | 1 | 2 | 4 | 7,7 | 1 |
| 46 | HFpEF | 4 | 4 | 4 | 8,4 | 1 | 4 | 4 | 7,8 | 1 | 4 | 4 | 8,2 | 1 |
| 47 | HFpEF | 3 | 0 | 3 | 7,1 | 0 | 0 | 3 | 7,6 | 0 | 0 | 3 | 7,6 | 1 |
| 48 | HFpEF | 3 | 0 | 3 | 7,1 | 0 | 0 | 3 | 7,4 | 0 | 0 | 3 | 7,7 | 1 |
| 49 | HFpEF | 3 | 0 | 3 | 8 | 0 | 0 | 3 | 7,5 | 0 | 0 | 3 | 7,6 | 1 |
| 50 | HFmrEF | 4 | 2 | 4 | 8,5 | 1 | 0 | 4 | 8 | 1 | 2 | 4 | 8,9 | 1 |
| 51 | HFmrEF | 4 | 1 | 4 | 6,9 | 1 | 0 | 4 | 7,4 | 1 | 1 | 4 | 7,1 | 1 |
| 52 | HFmrEF | 4 | 0 | 4 | 7,6 | 1 | 0 | 4 | 7 | 1 | 0 | 4 | 7,4 | 1 |
| 53 | HFmrEF | 3 | 2 | 3 | 7,3 | 1 | 2 | 3 | 7,1 | 0 | 2 | 3 | 7 | 0 |
| 54 | HFmrEF | 4 | 1 | 4 | 8,4 | 1 | 2 | 4 | 8 | 1 | 1 | 4 | 8,4 | 1 |
| 55 | HFmrEF | 3 | 1 | 3 | 7,3 | 0 | 2 | 3 | 6,8 | 0 | 1 | 3 | 7,4 | 0 |
| 56 | HFmrEF | 3 | 2 | 3 | 7,1 | 0 | 2 | 3 | 7,4 | 0 | 2 | 3 | 6,7 | 0 |
| 57 | HFmrEF | 4 | 1 | 4 | 7 | 0 | 1 | 4 | 7,2 | 0 | 1 | 3 | 6,6 | 0 |
| 58 | HFmrEF | 3 | 2 | 3 | 6,8 | 0 | 2 | 3 | 6,5 | 0 | 2 | 3 | 7 | 0 |
| 59 | HFmrEF | 4 | 2 | 4 | 8 | 1 | 2 | 4 | 7,6 | 0 | 2 | 4 | 7,9 | 0 |
| 60 | HFmrEF | 3 | 2 | 3 | 6,5 | 0 | 1 | 3 | 6,3 | 0 | 2 | 3 | 7 | 1 |
| 61 | HFmrEF | 4 | 2 | 4 | 8,8 | 1 | 2 | 4 | 8,4 | 1 | 2 | 4 | 8,5 | 1 |
| 62 | HFmrEF | 3 | 2 | 3 | 7,4 | 1 | 2 | 4 | 7,4 | 1 | 2 | 3 | 6,9 | 1 |
| 63 | HFmrEF | 4 | 1 | 4 | 7,5 | 0 | 1 | 4 | 7,7 | 1 | 1 | 4 | 8,1 | 1 |
| 64 | HFmrEF | 3 | 2 | 3 | 7,1 | 0 | 2 | 3 | 6,7 | 0 | 0 | 3 | 6,8 | 0 |
| 65 | HFmrEF | 3 | 1 | 3 | 7,6 | 0 | 1 | 3 | 7,4 | 1 | 2 | 3 | 7,9 | 0 |
| 66 | HFmrEF | 4 | 1 | 4 | 9,7 | 1 | 1 | 4 | 9,3 | 1 | 1 | 4 | 9,8 | 1 |
| 67 | HFmrEF | 4 | 1 | 4 | 7,2 | 1 | 1 | 4 | 7,4 | 1 | 1 | 4 | 7,9 | 1 |
| 68 | HFmrEF | 4 | 2 | 4 | 7,4 | 1 | 2 | 4 | 7,9 | 1 | 2 | 4 | 7,8 | 1 |
| 69 | HFmrEF | 3 | 2 | 3 | 6,8 | 0 | 2 | 3 | 7,3 | 0 | 2 | 3 | 6,3 | 0 |
| 70 | HFmrEF | 3 | 2 | 3 | 7,4 | 0 | 2 | 3 | 7,5 | 0 | 2 | 3 | 7,6 | 0 |
| 71 | HFmrEF | 3 | 2 | 3 | 8,3 | 1 | 2 | 3 | 8 | 1 | 2 | 3 | 7,7 | 1 |
| 72 | HFmrEF | 4 | 2 | 4 | 7 | 0 | 2 | 4 | 7 | 0 | 2 | 4 | 7,6 | 0 |
| 73 | HFmrEF | 4 | 1 | 4 | 6,5 | 1 | 1 | 4 | 7 | 1 | 1 | 4 | 6,1 | 1 |
| 74 | HFmrEF | 3 | 2 | 3 | 6,6 | 1 | 2 | 3 | 6,5 | 1 | 2 | 3 | 6,2 | 1 |
| 75 | HFmrEF | 3 | 1 | 3 | 6,8 | 0 | 1 | 3 | 7,3 | 0 | 1 | 3 | 7,2 | 0 |
| 76 | HFmrEF | 3 | 1 | 3 | 7,2 | 1 | 1 | 3 | 7,6 | 1 | 1 | 3 | 7,4 | 1 |
| 77 | HFmrEF | 3 | 2 | 3 | 7,7 | 0 | 1 | 3 | 7,2 | 0 | 2 | 3 | 7 | 0 |
| 78 | HFmrEF | 4 | 2 | 4 | 7,5 | 1 | 2 | 4 | 8,1 | 1 | 2 | 4 | 7,8 | 1 |
| 79 | HFmrEF | 4 | 1 | 4 | 7,8 | 1 | 1 | 4 | 8,4 | 1 | 1 | 4 | 7,8 | 1 |
| 80 | HFmrEF | 4 | 2 | 4 | 6,5 | 1 | 2 | 4 | 7,1 | 1 | 2 | 4 | 6,9 | 1 |
| 81 | HFmrEF | 3 | 2 | 3 | 7,7 | 1 | 2 | 3 | 7,9 | 1 | 2 | 3 | 8,3 | 1 |
| 82 | HFmrEF | 3 | 1 | 3 | 6,9 | 0 | 1 | 3 | 7,1 | 0 | 1 | 3 | 6,3 | 0 |
| 83 | HFmrEF | 3 | 1 | 3 | 6,6 | 0 | 1 | 3 | 6,7 | 0 | 1 | 3 | 6,2 | 0 |
| 84 | HFmrEF | 4 | 2 | 4 | 7,4 | 1 | 2 | 4 | 7,4 | 1 | 2 | 4 | 8 | 1 |
| 85 | HFmrEF | 3 | 1 | 3 | 6,6 | 0 | 1 | 3 | 7,2 | 0 | 1 | 3 | 7,1 | 0 |
| 86 | HFmrEF | 3 | 2 | 3 | 7 | 0 | 2 | 3 | 7,4 | 0 | 2 | 3 | 7,5 | 0 |
| 87 | HFmrEF | 3 | 2 | 3 | 7,6 | 1 | 2 | 3 | 7,1 | 1 | 2 | 3 | 7,2 | 1 |
| 88 | HFmrEF | 3 | 2 | 3 | 7,3 | 0 | 2 | 3 | 7,8 | 0 | 2 | 3 | 7,7 | 0 |
| 89 | HFmrEF | 3 | 2 | 4 | 8,3 | 0 | 2 | 3 | 7,7 | 0 | 2 | 3 | 7,8 | 0 |
| 90 | HFmrEF | 3 | 2 | 3 | 7,7 | 0 | 2 | 3 | 8,3 | 0 | 2 | 3 | 7,4 | 0 |
| 91 | HFmrEF | 4 | 2 | 4 | 7,8 | 0 | 2 | 4 | 7,4 | 0 | 2 | 4 | 7,3 | 1 |
| 92 | HFmrEF | 3 | 2 | 3 | 7,3 | 1 | 2 | 3 | 7,5 | 1 | 2 | 3 | 7,8 | 1 |
| 93 | HFmrEF | 4 | 2 | 4 | 8,7 | 1 | 2 | 4 | 8,9 | 1 | 2 | 4 | 8,7 | 1 |
| 94 | HFmrEF | 4 | 0 | 4 | 8,2 | 1 | 0 | 4 | 8,7 | 1 | 0 | 4 | 8,7 | 1 |
| 95 | HFmrEF | 3 | 2 | 3 | 6,3 | 0 | 2 | 3 | 7 | 0 | 2 | 3 | 6,5 | 0 |
| 96 | HFmrEF | 4 | 2 | 4 | 7,5 | 1 | 2 | 4 | 7,4 | 0 | 2 | 4 | 7,5 | 0 |
| 97 | HFmrEF | 4 | 2 | 4 | 9,1 | 1 | 1 | 4 | 8,6 | 1 | 2 | 4 | 8,7 | 1 |
| 98 | HFmrEF | 3 | 0 | 3 | 7,6 | 0 | 1 | 3 | 7,4 | 0 | 0 | 3 | 7,4 | 0 |
| 99 | HFrEF | 4 | 1 | 4 | 6,8 | 1 | 1 | 4 | 7,2 | 1 | 1 | 4 | 7,3 | 1 |
| 100 | HFrEF | 3 | 1 | 3 | 7,3 | 1 | 1 | 3 | 7,3 | 1 | 1 | 3 | 8 | 1 |
| 101 | HFrEF | 4 | 1 | 4 | 8 | 1 | 1 | 4 | 7,8 | 1 | 1 | 4 | 8,6 | 1 |
| 102 | HFrEF | 4 | 1 | 4 | 9,3 | 1 | 1 | 4 | 8,6 | 1 | 1 | 4 | 9 | 1 |
| 103 | HFrEF | 3 | 2 | 3 | 7,2 | 0 | 2 | 3 | 7,7 | 0 | 2 | 3 | 7,5 | 0 |
| 104 | HFrEF | 4 | 1 | 4 | 10,1 | 1 | 1 | 4 | 9,8 | 1 | 1 | 4 | 9,7 | 1 |
| 105 | HFrEF | 3 | 2 | 3 | 7,8 | 0 | 1 | 3 | 7,7 | 0 | 2 | 3 | 7 | 0 |
| 106 | HFrEF | 4 | 1 | 4 | 7,2 | 0 | 1 | 4 | 8,3 | 1 | 1 | 4 | 7,8 | 1 |
| 107 | HFrEF | 3 | 2 | 3 | 6,5 | 1 | 0 | 3 | 6,8 | 1 | 4 | 3 | 6,2 | 0 |
| 108 | HFrEF | 4 | 1 | 4 | 7,6 | 1 | 1 | 3 | 7,3 | 0 | 1 | 4 | 6,8 | 1 |
| 109 | HFrEF | 4 | 1 | 4 | 8,7 | 1 | 1 | 4 | 8,2 | 1 | 1 | 4 | 8 | 1 |
| 110 | HFrEF | 4 | 1 | 4 | 6,8 | 0 | 1 | 4 | 7,4 | 0 | 1 | 4 | 6,7 | 0 |
| 111 | HFrEF | 3 | 2 | 3 | 6,5 | 0 | 2 | 3 | 6,8 | 0 | 2 | 3 | 6,8 | 0 |
| 112 | HFrEF | 3 | 1 | 3 | 7,7 | 1 | 1 | 4 | 8,1 | 1 | 1 | 4 | 8,3 | 1 |
| 113 | HFrEF | 4 | 1 | 4 | 8 | 1 | 2 | 4 | 8,3 | 1 | 1 | 4 | 8,5 | 1 |
| 114 | HFrEF | 3 | 1 | 3 | 6,9 | 1 | 1 | 3 | 6,6 | 1 | 1 | 3 | 7,3 | 1 |
| 115 | HFrEF | 4 | 1 | 4 | 8,6 | 1 | 1 | 4 | 8,4 | 1 | 1 | 4 | 9,2 | 0 |
| 116 | HFrEF | 3 | 1 | 3 | 7 | 1 | 1 | 4 | 6,9 | 0 | 1 | 3 | 6,7 | 0 |
| 117 | HFrEF | 3 | 1 | 3 | 7 | 0 | 1 | 3 | 6,5 | 0 | 1 | 3 | 6,1 | 0 |
| 118 | HFrEF | 4 | 1 | 4 | 7,2 | 1 | 1 | 4 | 7,4 | 0 | 1 | 4 | 7,7 | 1 |
| 119 | HFrEF | 4 | 1 | 4 | 8,2 | 1 | 1 | 4 | 8,4 | 1 | 1 | 4 | 8,7 | 1 |
| 120 | HFrEF | 4 | 1 | 4 | 8 | 0 | 2 | 4 | 7,6 | 0 | 1 | 4 | 7,6 | 0 |
| 121 | HFrEF | 3 | 1 | 3 | 6,4 | 0 | 1 | 3 | 6,7 | 0 | 1 | 3 | 6,6 | 0 |
| 122 | HFrEF | 3 | 1 | 3 | 8,2 | 1 | 1 | 4 | 7,7 | 1 | 1 | 3 | 7,9 | 1 |
| 123 | HFrEF | 3 | 1 | 3 | 8,5 | 1 | 1 | 3 | 8,3 | 1 | 1 | 3 | 8,9 | 1 |
| 124 | HFrEF | 3 | 1 | 3 | 7 | 1 | 1 | 3 | 7,3 | 1 | 1 | 3 | 7,2 | 1 |
| 125 | HFrEF | 4 | 1 | 4 | 7,8 | 1 | 1 | 4 | 7,3 | 1 | 1 | 4 | 8,3 | 1 |
| 126 | HFrEF | 3 | 2 | 3 | 7,8 | 1 | 2 | 3 | 7,8 | 1 | 2 | 3 | 7,9 | 1 |
| 127 | HFrEF | 3 | 1 | 3 | 7,3 | 0 | 1 | 3 | 7,3 | 0 | 1 | 3 | 6,7 | 1 |
| 128 | HFrEF | 3 | 1 | 3 | 7,2 | 1 | 2 | 3 | 7,4 | 1 | 1 | 3 | 6,8 | 1 |
| 129 | HFrEF | 4 | 1 | 4 | 7,6 | 0 | 1 | 4 | 7,9 | 0 | 1 | 4 | 8,3 | 0 |
| 130 | HFrEF | 4 | 1 | 4 | 8,1 | 1 | 1 | 4 | 7,6 | 1 | 1 | 4 | 8,7 | 1 |
| 131 | HFrEF | 4 | 1 | 4 | 8,4 | 1 | 2 | 4 | 8 | 1 | 1 | 4 | 8,6 | 1 |
| 132 | HFrEF | 3 | 1 | 3 | 6,2 | 1 | 1 | 3 | 6,3 | 0 | 1 | 3 | 5,7 | 1 |
| 133 | HFrEF | 4 | 1 | 4 | 9,3 | 1 | 1 | 4 | 8,6 | 1 | 1 | 4 | 8,6 | 1 |
| 134 | HFrEF | 4 | 1 | 4 | 7,7 | 1 | 2 | 4 | 7,5 | 1 | 1 | 4 | 7,9 | 1 |
| 135 | HFrEF | 3 | 1 | 3 | 5,5 | 1 | 1 | 3 | 6,3 | 0 | 1 | 3 | 5,4 | 1 |
| 136 | HFrEF | 3 | 1 | 3 | 6,8 | 1 | 1 | 3 | 7,3 | 1 | 1 | 3 | 7,5 | 1 |
| 137 | HFrEF | 4 | 2 | 4 | 8,2 | 0 | 2 | 4 | 8,3 | 0 | 2 | 4 | 7,8 | 0 |
| 138 | HFrEF | 4 | 1 | 4 | 8,5 | 0 | 1 | 4 | 8,2 | 0 | 1 | 4 | 7,4 | 1 |
| 139 | HFrEF | 4 | 1 | 4 | 8,8 | 1 | 1 | 4 | 8,5 | 1 | 1 | 4 | 8,9 | 1 |
| 140 | HFrEF | 3 | 1 | 3 | 6,4 | 0 | 1 | 3 | 7 | 0 | 1 | 3 | 6,5 | 0 |
| 141 | HFrEF | 4 | 1 | 4 | 8,9 | 1 | 1 | 4 | 8,9 | 1 | 1 | 4 | 8,3 | 1 |
| 142 | HFrEF | 3 | 1 | 3 | 7,1 | 1 | 1 | 3 | 7,4 | 1 | 1 | 3 | 7,3 | 1 |
| 143 | HFrEF | 4 | 1 | 4 | 6,9 | 1 | 1 | 4 | 7,4 | 1 | 1 | 4 | 7,5 | 1 |
| 144 | HFrEF | 3 | 1 | 3 | 7,3 | 1 | 1 | 3 | 6,8 | 1 | 1 | 3 | 6,8 | 1 |
| 145 | HFrEF | 3 | 1 | 3 | 8 | 1 | 1 | 3 | 7,4 | 1 | 1 | 3 | 7,5 | 1 |
| 146 | HFrEF | 4 | 1 | 4 | 7,8 | 1 | 1 | 4 | 7,3 | 1 | 1 | 4 | 6,9 | 1 |
| 147 | HFrEF | 3 | 1 | 3 | 7,1 | 1 | 1 | 3 | 7,5 | 1 | 1 | 3 | 6,8 | 1 |
| 148 | HFrEF | 3 | 1 | 3 | 7,6 | 1 | 1 | 3 | 7,5 | 1 | 1 | 3 | 6,9 | 1 |
| 149 | HFrEF | 3 | 1 | 3 | 7,1 | 0 | 1 | 3 | 7,4 | 0 | 1 | 3 | 6,7 | 0 |
| 150 | HFrEF | 4 | 1 | 4 | 7,6 | 1 | 1 | 3 | 8,1 | 1 | 1 | 4 | 7,5 | 1 |
| 151 | HFrEF | 4 | 1 | 4 | 7,2 | 0 | 1 | 4 | 6,6 | 0 | 1 | 4 | 7,5 | 0 |
| 152 | HFrEF | 3 | 1 | 3 | 6 | 0 | 1 | 3 | 6,6 | 0 | 1 | 3 | 5,8 | 0 |
| 153 | HFrEF | 3 | 1 | 3 | 7,2 | 1 | 1 | 4 | 7,1 | 1 | 1 | 3 | 6,3 | 1 |
| 154 | HFrEF | 4 | 1 | 4 | 7,6 | 1 | 1 | 4 | 7,5 | 1 | 1 | 4 | 7 | 1 |
| 155 | HFrEF | 3 | 1 | 3 | 6,7 | 1 | 1 | 4 | 7,1 | 1 | 1 | 3 | 7,6 | 1 |
| 156 | HFrEF | 3 | 1 | 3 | 8,9 | 0 | 1 | 4 | 8,3 | 1 | 1 | 3 | 7,8 | 1 |
| 157 | HFrEF | 3 | 1 | 4 | 7,2 | 1 | 1 | 4 | 7,2 | 1 | 1 | 3 | 7,9 | 1 |
| 158 | HFrEF | 4 | 1 | 4 | 7,5 | 1 | 1 | 4 | 6,8 | 1 | 1 | 4 | 6,8 | 1 |
| 159 | HFrEF | 4 | 1 | 4 | 9,1 | 1 | 1 | 4 | 8,8 | 1 | 1 | 4 | 9,3 | 1 |
| 160 | HFrEF | 4 | 1 | 3 | 7,5 | 1 | 1 | 3 | 7,4 | 0 | 1 | 4 | 6,9 | 1 |
| 161 | HFrEF | 3 | 1 | 3 | 7,4 | 0 | 1 | 3 | 6,8 | 0 | 1 | 3 | 6,7 | 0 |
| 162 | HFrEF | 3 | 1 | 3 | 6,2 | 0 | 1 | 3 | 6,5 | 0 | 1 | 3 | 6,8 | 1 |
| 163 | HFrEF | 3 | 1 | 3 | 7,2 | 1 | 1 | 4 | 7,8 | 1 | 1 | 3 | 7,8 | 1 |
| 164 | HFrEF | 3 | 1 | 3 | 8 | 0 | 1 | 3 | 7,8 | 1 | 1 | 4 | 8,2 | 1 |
| 165 | HFrEF | 3 | 2 | 3 | 7,7 | 0 | 2 | 3 | 7,1 | 0 | 2 | 3 | 7 | 0 |
| 166 | HFrEF | 3 | 1 | 3 | 7,2 | 1 | 1 | 3 | 7,4 | 1 | 1 | 3 | 7,5 | 1 |
| 167 | HFrEF | 3 | 1 | 3 | 6,1 | 0 | 1 | 3 | 6,1 | 1 | 1 | 3 | 6,3 | 0 |
| 168 | HFrEF | 3 | 1 | 3 | 6,9 | 1 | 1 | 3 | 6,5 | 1 | 1 | 3 | 6,3 | 1 |
| 169 | HFrEF | 3 | 1 | 3 | 8,4 | 1 | 1 | 3 | 7,8 | 1 | 1 | 3 | 8,3 | 1 |
| 170 | HFrEF | 3 | 1 | 3 | 7,4 | 1 | 1 | 3 | 7,1 | 1 | 1 | 3 | 7 | 1 |
| 171 | HFrEF | 4 | 1 | 4 | 6,6 | 1 | 1 | 4 | 6,9 | 1 | 1 | 4 | 6,6 | 1 |
| 172 | HFrEF | 4 | 1 | 4 | 7,7 | 1 | 1 | 4 | 7,9 | 1 | 1 | 4 | 8,3 | 1 |
| 173 | HFrEF | 4 | 1 | 4 | 8,5 | 1 | 1 | 4 | 7,9 | 1 | 1 | 4 | 7,6 | 1 |
| 174 | HFrEF | 4 | 1 | 4 | 7,3 | 1 | 1 | 4 | 8,1 | 1 | 1 | 4 | 8 | 1 |
| 175 | HFrEF | 3 | 1 | 3 | 6,5 | 1 | 1 | 3 | 6,7 | 1 | 1 | 3 | 5,9 | 0 |
| 176 | HFrEF | 4 | 1 | 4 | 7,3 | 1 | 1 | 4 | 7,7 | 1 | 1 | 4 | 7,8 | 1 |
| 177 | HFrEF | 3 | 1 | 4 | 7,1 | 1 | 1 | 4 | 7,4 | 1 | 1 | 4 | 7,5 | 1 |
| 178 | HFrEF | 4 | 1 | 4 | 7,1 | 1 | 1 | 4 | 7,5 | 1 | 1 | 4 | 7,6 | 1 |
| 179 | HFrEF | 4 | 2 | 4 | 8,2 | 1 | 2 | 4 | 7,9 | 1 | 2 | 4 | 8,1 | 1 |
| 180 | HFrEF | 4 | 1 | 4 | 7,3 | 1 | 1 | 3 | 6,9 | 1 | 1 | 4 | 7,3 | 1 |
| 181 | HFrEF | 3 | 1 | 3 | 7,5 | 1 | 1 | 3 | 7,4 | 1 | 1 | 3 | 7,9 | 1 |
| 182 | HFrEF | 4 | 1 | 4 | 8 | 1 | 1 | 4 | 7,8 | 1 | 1 | 4 | 8,4 | 1 |
| 183 | HFrEF | 4 | 1 | 4 | 8,4 | 1 | 1 | 4 | 7,8 | 1 | 1 | 4 | 7,9 | 1 |
| 184 | HFrEF | 4 | 1 | 4 | 8,4 | 1 | 1 | 4 | 7,9 | 1 | 1 | 4 | 8,4 | 1 |
| 185 | HFrEF | 4 | 1 | 4 | 8 | 1 | 1 | 4 | 7,5 | 1 | 1 | 4 | 7,7 | 1 |
| 186 | HFrEF | 4 | 1 | 4 | 6,6 | 1 | 1 | 4 | 6,9 | 1 | 1 | 4 | 6,9 | 1 |
| 187 | HFrEF | 3 | 1 | 3 | 7,4 | 1 | 1 | 3 | 7,9 | 1 | 1 | 3 | 8 | 1 |
| 188 | HFrEF | 4 | 1 | 4 | 7,2 | 0 | 1 | 4 | 7,7 | 1 | 1 | 4 | 6,4 | 0 |
| 189 | HFrEF | 3 | 1 | 4 | 8,5 | 1 | 1 | 4 | 8,1 | 1 | 1 | 4 | 8,7 | 1 |
| 190 | HFrEF | 4 | 1 | 4 | 8,5 | 1 | 1 | 4 | 7,7 | 1 | 1 | 4 | 7,7 | 1 |
| 191 | HFrEF | 4 | 1 | 4 | 8,9 | 1 | 1 | 4 | 8,6 | 1 | 1 | 4 | 9 | 1 |
| 192 | HFrEF | 3 | 1 | 3 | 6,6 | 1 | 1 | 3 | 6,8 | 1 | 1 | 3 | 6,5 | 1 |
| 193 | HFrEF | 4 | 1 | 4 | 7,6 | 1 | 1 | 4 | 7,7 | 1 | 1 | 4 | 8 | 1 |
| 194 | HFrEF | 4 | 1 | 4 | 9 | 0 | 1 | 4 | 8,6 | 1 | 1 | 4 | 8,4 | 0 |
| 195 | HFrEF | 3 | 1 | 3 | 7,1 | 1 | 1 | 3 | 7,1 | 1 | 1 | 3 | 7 | 1 |
| 196 | HFrEF | 3 | 1 | 3 | 7,2 | 0 | 1 | 3 | 7,2 | 0 | 1 | 3 | 6,7 | 0 |

*0 = atrial etiology, 1 = ventricular etiology, 2 = atrioventricular etiology, 3 = primary etiology, 4 = mixed etiology

**Supplemental Figure 1: Bland Altman Plots:** Bland Altman plots for agreement of Vena contracta measurements for inter- and intraobserver variability for each HF subgroup

**Supplemental Figure 2: Univariate Cox regression forest plot.** Association of all clinical, echocardiographic and laboratory variables with mortality, depicted by HRs and 95% Cis (derivation cohort); BUN=blood urea nitrogen; GOT= Aspartate transaminase, GPT= Alanine transaminase, CRP=C-reactive protein, GGT= γ-Glutamyl transferase, AFIB=atrial fibrillation; Nt-proBNP=N-terminal *pro* brain-type natriuretic peptide; COPD = chronic obstructive pulmonary disease, CAD=coronary artery disease;

**Supplemental Figure 3: Stepwise bootstrapped selection.** Stepwise bootstrap resampling procedure identifying the most comprehensive set of variables associated with mortality among (i) all clinical parameters (ii) all echocardiographic variables and (iii) all laboratory variables (derivation cohort). BUN=blood urea nitrogen; GOT= Aspartate transaminase, GPT= Alanine transaminase, CRP=C-reactive protein, GGT= γ-Glutamyl transferase, AFIB=atrial fibrillation; Nt-proBNP=N-terminal *pro* brain-type natriuretic peptide; COPD = chronic obstructive pulmonary disease, CAD=coronary artery disease;

**Supplemental Figure 4: Kaplan-Meier analysis of identified subgroups in validation cohort.** Subgroups are color coded according to their survival, patients in subgroup 7 (dark blue) had the most favorable survival, while subgroup 5 (darkred) displayed excess mortality.


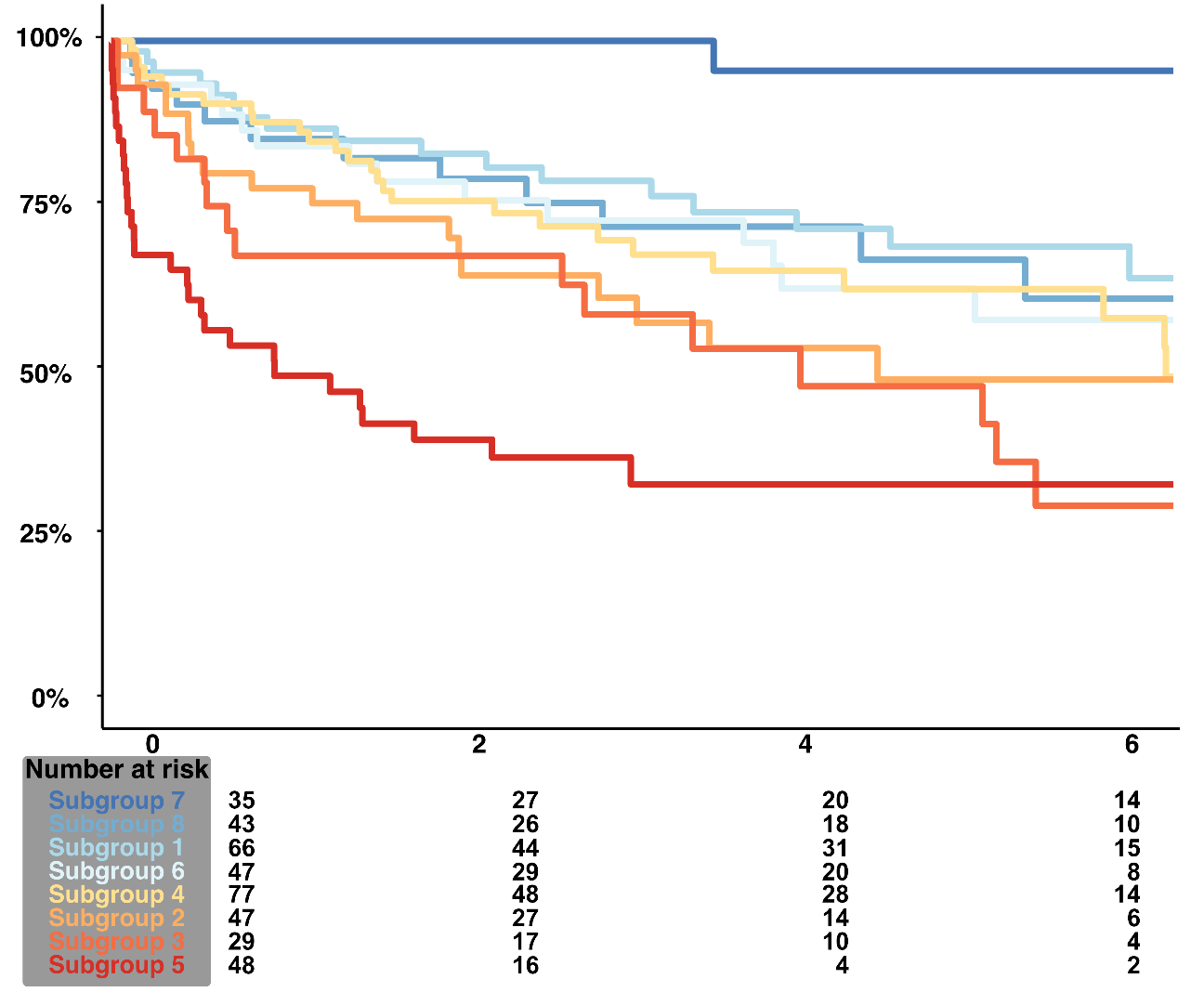


**Supplemental Figure 5: Temporal validation**. Patients were pooled into 2 groups by year of echocardiogram to check consistency of echocardiograms over the study period. Both year groups show similar differentiation of identified subgroups

**Supplemental Figure 6:** Forestplot of univariate Cox Regression depicting HRs and 95% Cis for all clinical, echocardiographic and laboratory predictors according to heart failure type. HFpEF=heart failure with preserved ejection fraction (>50%). HFmrEF= heart failure with mid-range ejection fraction (40-50%). HFrEF=heart failure with reduced ejection fraction (<40%). BUN=blood urea nitrogen. AFIB=atrial fibrillation. HB=hemoglobin Nt-proBNP=N-terminal pro brain-type natriuretic peptide. (subgroup analysis).

**Supplemental Figure 7:** Boostrapped stepwise selection of clinical, echocardiographic and laboratory predictors after 500 repeats for each heart failure subtype. (subgroup analysis).

**Supplemental Figure 8:** Survival tree and according Kaplan Meier curves for HFpEF patients. The subgroup with the most favorable survival is colored red and depicted in every Kaplan Meier curve for reference (subgroup analysis).

**Supplementary** **Figure 9:** Survival tree and according Kaplan Meier curves for HFmrEF patients. The subgroup with the most favorable survival is colored red and depicted in every Kaplan Meier curve for reference (subgroup analysis).

 **Supplemental Figure 10:** Survival tree and according Kaplan Meier curves for HFrEF patients. The subgroup with the most favorable survival is colored red and depicted in every Kaplan Meier curve for reference (subgroup analysis).
